# Examining the fluidity of innovation teams: a conceptual framework

**DOI:** 10.3389/fpsyg.2023.1296651

**Published:** 2023-12-18

**Authors:** Rylee M. Linhardt, Eduardo Salas

**Affiliations:** Department of Psychological Sciences, Rice University, Houston, TX, United States

**Keywords:** fluid teams, knowledge integration, team innovation, team resilience, mutual trust, team reflexivity

## Abstract

As innovative endeavors have become more complex and time-intensive, there has become an increasing reliance on expert teams in organizations. Expert innovation teams are comprised of team members with extensive experience and mastery in a particular discipline. These teams utilize fluid membership that expands the available knowledge of the team but creates challenges for effective teamwork. We argue that the mechanism for creating an enduring impact and developing a product to fruition requires the cognitive and social integration of fluid team members. This article focuses on how teams effectively integrate knowledge with diverse, and possibly fluid, team members and how teams can organize knowledge through planning and reflection to implement the idea successfully. Knowledge integration and team reflexivity are considered in tandem to emphasize the multi-faceted nature of generating and implementing innovative solutions and the conflicting teamwork processes that hinder innovative efforts. To understand how these competing teamwork processes required for successful innovation interact, we developed a framework that considers resilience as the factor that elicits team creative performance. In doing so, we discuss how innovation teams build resilience over time and how creative failure can lead to greater levels of innovation.

## Introduction

Advancements in technology have created a desire for entrepreneurs to develop innovative products and services at a greater magnitude. More large-scale and sophisticated innovation is often considered a global effort where actors allocate a substantial amount of resources to a goal that has yet to be achieved by previous innovators. In the early years of the space industry, as one example, innovation was inherently risky and prone to failure because there was no foundation of knowledge to achieve such goals (Horais, 2016). Today, although equally risky, the space industry is refining the current standard from previous engineering efforts, much like the aviation industry in the 1950s and 60s, by commercializing space travel and making it more sustainable and efficient to implement.

Innovation in the space industry is a multi-team effort where expert teams collaborate to effectively overcome complex and novel problems. Projects that are currently being pursued (e.g., Blue Origins New Glenn; SpaceX Vast Haven-1; James Webb Space Telescope) require such complex teaming because of the intricate and comprehensive nature of the task. Another implication of innovation is the need to outsource knowledge to individuals with specific expertise and backgrounds that contribute to specific innovative challenges. Fluid membership is an additive complexity of innovation teams because it disrupts the teamwork processes necessary to be creative at a time when fluid team members’ expertise is needed to resolve creative limitations or restrictions. In other words, when a fluid member becomes a part of the team’s knowledge composition, the change in composition is likely because weaknesses in the team’s knowledge and skills cannot overcome the current constraints of the innovative environment. Therefore, the additive effects of fluid membership and the interdependent nature of the cognitive processes make it difficult for innovation teams to generate and implement novel ideas.

This has created a need to understand how teams with expert knowledge can effectively coordinate efforts within and across teams to deliver a functional product. To be successful, a high level of knowledge integration is required due to the nature of innovative projects. Similarly, as a consequence of the time dedicated to achieving complex innovation, innovation teams must engage in detailed planning and reflection so knowledge is not lost as teams pursue different ideas and implementation strategies. As teams work through innovative problems and have developed an organized knowledge structure, they can integrate more knowledge using a complex and dynamic collective understanding from previous team knowledge. Teams that have developed an integrated collective understanding have a more organized knowledge structure due to the effective planning and reflection that connects how and why a creative idea failed or succeeded. Teams can use the information from explicit planning to build a more comprehensive collective understanding made up of team members’ expertise that, in combination and with extensive evaluation by expert team members, leads to a higher degree of innovation. In the following paper, we identify the teamwork processes that elicit more innovative behavior while also considering the challenges, mainly fluid membership, innovation teams must face as they generate and implement creative ideas.

**Figure 1:**
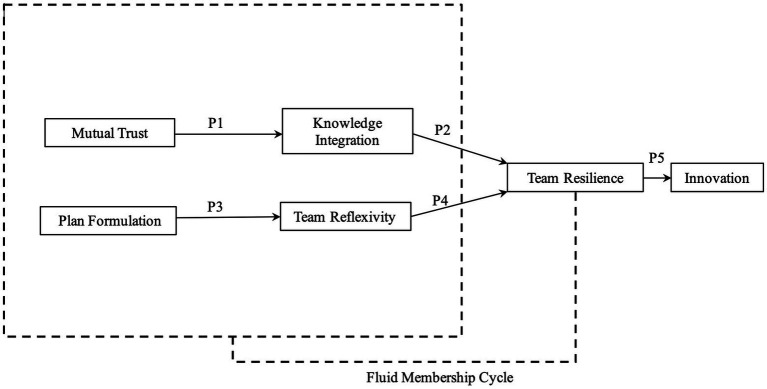
Conceptual framework of fluid membership in innovation teams.

The aim of this paper is to understand the teamwork processes that help coordinate diverse expertise in fluid innovation teams. In doing so, we first address the knowledge integration needed to generate novel solutions and how fluid membership can enhance innovation teams’ collective understanding. We do so by considering mutual trust as a driver of knowledge transfer and integration between team members leading to more novel solutions. Second, we address the challenges innovation teams have with coordinating diverse expertise in an ill-defined environment and the disruption of teamwork processes due to fluid membership. Planning for effective coordination can help determine when fluid membership is necessary or if certain expertise is needed at a later point in the creative project. We argue that planning is a mechanism teams can use to overcome diverse fluid membership and reflect on teamwork processes that were attributed to creative success or failure. We attribute mutual trust and effective planning to a team’s ability to generate innovative solutions by developing their collective understanding and shared mental model of the teamwork processes necessary for team-level creative problem-solving. To further understand the teamwork processes that prompt team innovation we offer a framework (Figure 1) and accompanying propositions that illustrate how teams can effectively coordinate and build resilience to adverse events as they progress with innovative efforts. We propose that as teams collectively experience failure, the team will become more resilient to unexpected and novel problems through effective coordination throughout the creative process.

## Conceptual development of framework

### Fluidity and team innovation

Innovative organizations find fluid membership attractive because teams can be flexible to rapidly changing contextual demands of implementing an innovative idea (Schreyögg and Sydow, 2010; Huckman and Staats, 2011; Backmann et al., 2015). New product design teams often used for large-scale innovation in the space industry are designed in a matrix format where a team of leaders oversees more specialized teams (Kratzer et al., 2010). These specialized design teams solve problems related to their team’s expertise that often inhibit the larger creative goal from being successfully implemented. This type of innovation team (i.e., new venture teams, entrepreneurial teams, new product design teams, X-teams) requires team members of different backgrounds and expertise to collaborate effectively to ensure the requirements are met for the overarching creative goals across teams (Quinn and Mueller, 1963; Burgleman, 1983; Ancona and Caldwell, 1987, 1992). The challenges of reaching successful innovation include not only generating novel solutions but also ensuring they do not obstruct other teams’ progress toward the same creative goal. When these specialized teams find solutions that meet the overarching creative goals and those of other teams, the novel solutions can then be pieced together by the leadership team resulting in dramatic innovation.

Innovative organizations and innovation teams that use this team structure often exist until the innovative product is delivered. Until the fruition of the project, however, innovation teams experience team composition change, like typical teams, but can leverage unstable membership and outside resources when unexpected problems arise that require specialized knowledge (Huckman et al., 2009). Fluidity has been largely studied in aversive contexts like health care (e.g., Bedwell et al., 2012) and emergency teams (Cristancho et al., 2022) but also in teams where problems are extremely complex and prone to failure. Fluid teams are drawn from a problem-centered approach in healthcare where multidisciplinary employees coordinate around the needs of the patient (Bleakley, 2014). Similar to healthcare, fluidity in emergency teams (e.g., military teams, first-responder teams) is derived from the environmental and situational cues that require other team members to assist in resolving a crisis or critical incident. Fluidity in these contexts is viewed as a method of addressing and resolving current situations and is not used to create and build knowledge in a team. Some literature has shown fluid membership to be effective in innovative environments because team members can interact with others to create new combinations and build a collective understanding of knowledge that results in innovation (Edmondson and Nembhard, 2009; Salazar et al., 2012).

Creativity and innovation are often used interchangeably in the literature (Zhou and Hoever, 2014). However, the distinction between creativity and innovation is important to recognize for teams engaging in complex and ill-defined problems. Innovation in an organizational setting is characterized by a dynamic, competitive, and often expensive environment (Scott and Bruce, 1994). To successfully carry out larger and more complex innovation, organizations employ teams of specialized technical experts to generate novel ideas collectively and develop a strategy to successfully implement the idea (Im et al., 2013). Importantly, creative ideas operating in ill-defined environments are often prone to failure and risky to implement at the team (Hülsheger et al., 2009; Edmondson, 2013) and organizational levels (Frambach and Schillewaert, 2002). For this reason, innovation teams are more successful when they integrate team members’ diverse expertise that, in combination, leads to more novel and implementable solutions.

Novelty and usefulness are core elements of innovation (Shalley, 1991; Amabile, 1996; Ford, 1996; Oldham and Cummings, 1996; Zhou, 1998). For creative ideas to be successful, they need to be both novel and useful, where the idea can be successfully implemented. Innovation is considered a multi-step process that is interdependent and dynamic yet involves conflicting cognitive and teamwork processes for teams engaging with a novel problem. The processes that are essential for team-level innovation more broadly include problem construction, idea generation, and idea evaluation (Reiter-Palmon et al., 2008; Leone and Reiter-Palmon, 2022). Several theoretical conceptualizations of the team innovation process suggest these processes are dynamic where teams move forward and refer back to the earlier creative process to resolve problems and limitations as the idea comes to fruition (Nonaka, 1994; Stenmark et al., 2011).

Innovation teams are more successful when provided with a clear understanding of the tasks and goals of the project early in the innovation process (Mumford et al., 1994). Problem construction, and subsequent problem conceptualization, can benefit creative ideas because teams can structure knowledge to become more familiar with the problem before generating or evaluating the idea (Mumford et al., 1991; Reiter-Palmon and Murugavel, 2018). At the team level, team members can engage in problem construction or conceptualization together, so they can integrate their expertise and structure team knowledge effectively later in the creative process (Salazar et al., 2012). As teams conceptualize the problem, they develop an individual understanding first, which is then integrated and reconfigured collectively based on the fragmented but overlapping representations of team member’s expertise.

### Knowledge integration

Although problem construction is considered a critical innovative process, it does not translate into innovation independently (Somech and Drach-Zahavy, 2013; Wu et al., 2022). Problem construction can lead to more effective idea generation in teams and can be expanded upon as the team engages more in that particular area of expertise (Reiter-Palmon, 2017). Idea generation is more prevalent in the early stages of the project and initially while evaluating ideas (West, 2002). The purpose of idea generation for teams is to broadly conceive ideas that *may or may not* lead to a creative solution using their collective understanding of the problem (Mumford et al., 2012). As these ideas are parsed through for their usefulness, information that is derived from these evaluations can be used to further construct the problem or generate ideas that further refine the idea.

Often, team members generate ideas independently and then discuss them at large with the team to find connections within the team’s expert knowledge (Stroebe and Diehl, 1994). The team’s collective understanding at the earlier stages of the creative process is more malleable and considers the knowledge created from the plethora of ideas generated by team members. Teams may generate many ideas that the team has a surface-level understanding of when evaluating each idea to become more knowledgeable about the ideas worth pursuing. This approach to team idea generation has been shown to be effective for the number of ideas generated by the team (Paulus and Yang, 2000). To be successful, innovation teams must balance generating novel ideas that are unrestricted and evaluating the idea for it to be implementable. A large number of the initial ideas are ultimately discarded when the ideas are vetted by the team for their implementability (Kennel et al., 2013). The ideas that are ultimately not used can be helpful in building a team’s collective understanding and may be useful later on in the project. Similarly, these discarded ideas could potentially be combined with new expert knowledge, but must be accessible for the team to use at different points in the creative process.

In teams with diverse expertise, idea evaluation is critical in determining the success of the idea (Licuanan et al., 2007; McCarthy et al., 2018). When teams evaluate ideas conjunctively, they can determine the restrictions that will cause the idea to fail from different perspectives. In a similar fashion, team members can integrate these ideas together in concert with diverse perspectives that help to overcome the constraints hindering innovation (Fleming and Sorenson, 2004). As teams generate and integrate novel solutions over time, team members can build upon an already existing knowledge structure rather than generating entirely new ideas (Garud and Nayyar, 1994). When the knowledge structures are expanded upon over time using diverse expertise it will broaden the range of knowledge that can be integrated into the team’s collective understanding. This is contingent, however, on the ability of the team to sustain their collective understanding until the creative idea is successfully implemented and also integrate new knowledge effectively at each stage of the creative process even if ideas were generated at different points in time.

The integration of knowledge is inherently linked with building a collective understanding of the environment, problem, and potential solutions. With each success or failure of a creative idea in the scheme of a larger creative goal, team members develop a better conceptualization of the problem and concepts that lead to successful outcomes. The diverse representations of the environment and conceptualization of the problem from team members can be used to further appraise the idea as a collective to generate new knowledge. For instance, evaluations of diverse team members’ ideas can help to consider new knowledge and assimilate it into the current collective understanding. This collective understanding can be further built upon as the team continues to revise ideas with different combinations of diverse, and possibly fluid, team members. When the team has a collective understanding that includes insights from different disciplines, the team can then connect concepts from different potential solutions that together overcome the novelty and complexity of the problem.

However, innovation teams face specific challenges when integrating knowledge from team members with deep knowledge of their discipline. The disciplines and sub-disciplines that give team members unique knowledge dictate how individual team members will conceptualize the problem (Eigenbrode et al., 2007). When teams are comprised of diverse technical experts, team members create different mental models that may differ dramatically within the innovation team, so much so the ideas and how they are implemented differ drastically (Davison and Blackman, 2005). When the mental models of team members differ greatly, teams cannot work interdependently to combine knowledge and build a collective understanding effectively. It is essential that team members’ mental models converge to build a collective and dynamic knowledge structure that supports novelty but also prescriptions to implement ideas that converge between team members. Salazar et al. (2012) describe a team’s integrative capacity as their ability to develop a sustainable integrative system of social, psychological, and cognitive processes that facilitates team members combining distinct expertise and working as a unified whole (Balakrishnan et al., 2011). A team’s integrative capacity helps the team to develop their shared mental model of the creative domain, which includes how the team will interact for knowledge to be effectively transferred between team members (Cannon-Bowers et al., 1993; Mathieu et al., 2000; DeChurch and Mesmer-Magnus, 2010). Salas et al. (2005) described a team’s shared mental model as “an organizing knowledge structure of the relationships among the task the team is engaged in and how the team members will interact.” A team’s integrative capacity and shared mental model relate because together both enhance a team’s vision and strengthen the co-creation process between team members (Hensel and Visser, 2019).

With a more integrated shared mental model, the team can interpret ill-defined changes more easily (Cannon-Bowers et al., 1993), but also anticipate the needs of both fluid and diverse members (DeChurch and Mesmer-Magnus, 2010) and efficiently coordinate with others when a novel problem arises (Mohammed and Dumville, 2001). When fluid membership is necessary to refine a creative idea, the team’s shared mental model can facilitate both cognitive and interpersonal integration. Thus, with each respective disruption, likely including both fluid membership and task change, the team’s shared mental model could expand if the fluid team members’ expertise aligns with stable members, so they can interact and transfer expert knowledge.

For innovation teams, knowledge from outside sources supplies new information for the team to generate novel solutions. When the complexity of the problem exceeds what is currently available for the current team, fluid membership can provide the specialized knowledge necessary to aid in building a more dynamic collective understanding (Bushe and Chu, 2011). More effective knowledge integration occurs when (new) expertise is combined (recombined) with the team’s current knowledge structure through collective evaluation (Garud and Nayyar, 1994; Fleming, 2001; Fleming and Sorenson, 2004). In other words, innovation teams find a rhythm when evaluating and revising successful creative solutions and can resolve similar problems that require expertise that is analogous to the problems resolved previously. However, another challenge innovation teams face is when unexpected or novel problems arise that may differ greatly from previous team experiences and require a different combination of team members’ expertise. When the team shifts efforts due to a novel problem, prompting fluid membership, the team will face disruptions to not only the information flows but also teamwork processes that help team members generate novel solutions collectively (Sosa et al., 2004; Wallace et al., 2004; Sosa, 2008; Rai et al., 2009; Gokpinar et al., 2010).

The interpersonal processes of innovation teams are critical for teams that generate ideas independently (or collaboratively) and collectively evaluate them. The interpersonal processes affect idea evaluation when team members have developed relationships with others on the team so they, together, can extensively evaluate and generate new ideas based on their collective appraisal. The connection between team members goes beyond just assessing and identifying diverse knowledge in innovation teams. Team members have to combine concepts that are novel in an already complex environment, in which interpersonal connections with team members with the same goal are salient to the knowledge integration that leads to innovation. Prosocial motivation, or team members prioritizing team outcomes over individual outcomes, is the boundary between teams integrating knowledge and achieving high levels of creativity (Wu et al., 2022). For teams to develop a strong integrative capacity, team members must heavily consider different ideas and trust the expertise and motivations of others working toward the team’s creative goals.

### Mutual trust

There are few disadvantages to diverse intelligence in expert teams when team members’ efforts to build a collective understanding include a shared vision of the innovative idea (O’Kane, 2017). A shared vision can indicate to diverse team members that the purpose of knowledge sharing and integration is to achieve the collective goal of implementing the innovative idea. Innovation teams often have an inflated sense of vision around the success of the creative idea (Logue and Grimes, 2022) which can motivate teams to see a creative idea to fruition and share the same goals. This vision also motivates team members to avoid interpersonal conflict for the sake of integrating knowledge, increasing the likelihood that team members will consider others’ ideas (Majchrzak et al., 2012). When evaluating ideas, team members who share a similar vision may view others’ evaluations as less threatening and are more willing to pursue a novel idea in combination with their expertise. However, a team’s shared vision, along with their shared mental model, may be adversely impacted by fluid members who are not familiar with the idea and who have not built relationships with other team members. Importantly, for innovation teams, social integration and cognitive processes must coevolve to build the mutual trust that leads to more knowledge integration (Wang et al., 2006; van Knippenberg, 2017). In other words, the constant flow of new team members may disrupt the social processes that need to evolve with the development of a team’s collective understanding, inhibiting the degree of knowledge considered when the team is refining their knowledge structure.

The social processes are disrupted because fluid members are inherently less committed to the team or idea (McCarthy et al., 2019). Lower commitment can be positive for the team given that fluid team members are not committed to a particular idea or process and are more willing to shift efforts or consider other ideas. Stable team members, on the other hand, who have a salient vision and who have devoted more time to the project, are less likely to accept change, especially from a team member who does not have a similar level of project knowledge and experience (Bushe and Chu, 2011). Considering stable members have a strong vision and team commitment, integrating new knowledge from unfamiliar team members that deviate from the previous strategies may be difficult. For the fluid team members beginning to work with stable team members, rigorously evaluating novel ideas and voice evaluations that go against the team’s collective understanding might not be straightforward.

For fluid team members to evaluate and refine ideas more frequently and intensely, they need to create a psychologically safe environment (West, 1990; Edmondson, 2003; West, 2003; Widmer et al., 2009; Wei et al., 2023). When difficult conversations or conflicts emerge from different team members’ expertise and team status, psychological safety can help them collaborate to find consensus when refining the idea. Psychological safety opens team members up to novel ideas from different team members, inclining those team members to share unique and possibly useful ideas that can extend the team’s collective understanding to an innovative idea. When innovation teams become less resistant to changing the idea, fluid team members not only feel safer sharing perceptions or evaluations, but stable team members are open to refining the idea (Wall and Lischeron, 1977; West, 1990). Therefore, fluid team members offer a new and improved perspective when evaluating ideas and do so to a greater degree than if psychological safety was not present.

Evaluations based on different perspectives of team members in a psychologically safe environment can help innovation teams disseminate concerns or discuss potential errors. Teams that share knowledge more, especially when team members are diverse, will likely extend that knowledge into new innovative ideas or solutions. By integrating knowledge, innovation teams are addressing a potential problem that has not been identified under the team’s current knowledge base or capabilities. When novel perspectives are introduced that expand the idea or address a problem, new ideas emerge that are a collective of novel knowledge and already established dynamic collective understanding. As new ideas emerge, with the help of fluid team members’ contributions, surface-level weaknesses are addressed that could advance into detrimental flaws later in the creative process (Edmondson and Lei, 2014). When problems are addressed earlier, the problems can be accounted for as team members integrate their expertise. The more critically novel the evaluations become using different combinations of knowledge, the team’s collective understanding is further refined but includes an expanded understanding of how the idea meets innovative standards.

As psychological safety encourages more dynamic evaluations and reflections of current ideas the fluid and stable team members begin to trust in each other’s judgments and decision-making. With greater mutual trust between team members, fluid and stable team members can begin to oppose and be vocal in their dissent of assumptions or long-standing beliefs of the team’s collective understanding. For fluid team members, dissent is connected to expertise that has not been applied to the current collective understanding, to which the stable team members can evaluate and determine if it furthers the knowledge needed to find creative solutions. This allows for stable members to alter how they perceive the situation in relation to their expertise and where they can apply their novel ideas to the revised collective understanding. The result of this comparison creates a consensus between team members that is different from what was previously considered as a part of the original collective understanding. Importantly, the team’s new collective understanding is structured based on the novel contributions, but also the limitations, of both expert perspectives.

To encourage critical evaluation and knowledge integration, both fluid and stable members need to trust in the capabilities of themselves and other team members. Mishra (1996) described trust as “the willingness to be vulnerable to another party based on the others being reliable, competent, open, and concerned about one another.” The mutual trust provided by a psychologically safe environment, especially between fluid and stable members, augments the degree of knowledge sharing and integration between team members, which advances the depth of cognitive integration and enhances interpersonal relationships (Rauniar et al., 2019; Zahoor et al., 2021). Considering the degree of complexity and ambiguity of the problem, the beliefs surrounding a person’s competence influence how much they will trust that team member to carry out difficult tasks successfully (Kianto, 2011).

Similarly, ideas from competent team members may be more heavily considered, and team members will trust in the idea for longer, multiplying the depth of knowledge integration. When team members trust each other’s abilities, team members are more willing to generate novel ideas collectively and collaborate to reach the next phase of implementing the idea (Hargadon and Bechky, 2006). More specifically, fluid and stable members who trust in each other’s skills and expertise can contribute to the collective understanding, but more importantly, team members can collaborate to combine novel expertise that can lead to more innovative ideas. Team members who trust each other are more willing to consider others’ ideas more quickly and refine them using their expertise to determine if it is a creative solution (Barczak et al., 2010).

Paired with a shared vision, mutual trust is essential for innovation teams to interact and exchange information but is hindered by fluid membership (Dineen and Noe, 2003; Noe et al., 2003; Sonnenwald, 2007; Rauniar et al., 2019). Fluid members need to trust in the shared vision of the stable team members, so fluid members are motivated to contribute knowledge to the team’s collective understanding. More stable members need to trust that newcomers will add to the conceptual knowledge of the team and are competent in their discipline. When mutual trust exists, fluid and stable team members can socially integrate better and build the necessary communication networks to create knowledge through cognitive integration (Rauniar et al., 2019). Knowledge integration and mutual trust relate to how a team’s creative solutions can be further refined through the diverse expertise of fluid membership leading to larger-scale innovation.

*Proposition 1: Mutual trust will strengthen the capacity of fluid members and stable members in innovation teams to integrate knowledge leading to more novel solutions.*

### Resilience

Mutual trust is necessary for innovation teams and is a mechanism to effectively build resilience toward innovative challenges (Bowers et al., 2017; Varajão et al., 2020; Bisbey et al., 2021). Considering the prolonged period of time teams are working in an innovative environment and the pressure to deliver a usable product, innovation teams will experience error and taskwork failures (Shawn Burke et al., 2005). Teams who experience failure and use it as a learning opportunity will create positive adaptations to the teamwork processes and knowledge that caused the critical failure (Gucciardi et al., 2018). At each reflection point, the team can further understand why and how team member interactions and knowledge integration contributed to better or worse team innovation. Innovation teams can then reconsider their actions, while planning and reflecting, and change them prior to a new fluid membership cycle so ineffective teamwork processes aren’t carried over to the new, and moldable, team dynamic. While the team is engaging in planning and reflection team members are making sense of why the previous failure occurred and how the team’s actions lead to that outcome. Over time, the team will gain more experience working together and learn how to effectively navigate the ill-defined environment while being innovative through teamwork and team cognition.

While the team is planning and reflecting, they should engage in convergent thinking and align in a direction using their new shared understanding. However, given expert team members have a different interpretation of why and how the creative idea ended in failure or was successful, suggesting reflection may be crucial for teams to develop an innovative shared interpretation (Bellis and Verganti, 2021). The interplay between the different interpretations lends to divergent thinking, where the interpretations of team members are identified. Identifying different perspectives can then be used to engage in more collective convergent thinking where the team can make sense of why and how the team arrived at this point in the project. Team members collectively discussing and resolving conflicting perspectives in a psychologically safe environment *make sense* of other’s perspectives which drives consensus and a shared understanding of the current knowledge structure (Berger and Luckmann, 1966; Goodman, 1978).

As the team members are sensemaking together, team members move towards a more refined collective understanding. Sensemaking can be interpreted as an organizing activity of what is occurring at a particular point in the innovative project (Talat and Riaz, 2020). The more complex the innovative idea, the more sensemaking is needed at each step of the innovative process. Sensemaking becomes essential when many different interpretations are being considered, so teams can determine what is relevant to expanding their collective understanding. The important aspects derived from sensemaking can be retained into the team’s collective understanding, which can then be used for future engagement with the creative idea. While the collective understanding is being updated, the team is also developing a shared understanding of what knowledge should be applied to future team creative processes and ideas. When mutual trust exists within the team, team members shared mental model, including their shared understanding and consensus on how the team should engage in teamwork processes, will persist toward a shared representation of the problem. Reflection and planning processes strengthen the teams’ transactive memory system (TMS), where knowledge is organized within a respective period of the innovative idea. Therefore, when specific past knowledge aids in a future innovative problem, it has been organized in a way that is more accessible for new team members to apply to their collective understanding.

Researchers have considered team resilience in the context of high-stakes industries and have found resilience to be fundamental for the team to maintain composure under pressure, recover from adverse events, and adapt to ill-defined situations (Alliger et al., 2015; Bowers et al., 2017; Bisbey et al., 2021). Although there is still a need for consensus in defining team resilience, Alliger et al. (2015) conceptualized team resilience as “the capacity of a team to withstand and overcome stressors in a manner that enables sustained performance” (see Chapman et al., 2020). As team members interact, team resilience will emerge and evolve over time (Bowers et al., 2017). This suggests the capacity for innovation teams to be resilient will accumulate and shift (positively or negatively) with each adverse event.

Challenging events and high-risk problem-solving place stress on both the cognitive and interpersonal processes of innovation teams, testing the ability of the team to bounce back from these challenges and move forward with the innovative idea. When innovation teams are not resilient to these chronic challenges, team members consider their individual goals over team goals, ultimately weakening their sense of collective vision around the idea. As the teamwork processes diminish and team members become more individualistic, motivation to combine knowledge and coordinate those efforts effectively is reduced. As the team moves forward with innovative efforts, their collective understanding will not expand to new domains of expertise because of the lack of knowledge integration by expert team members. Importantly, less socialization earlier in the process will impact the team later in the creative process when they integrate knowledge because they have not established or maintained the interpersonal processes necessary to integrate expertise effectively.

These effects could be exacerbated for fluid team members who could become hesitate to voice concerns about novel knowledge combinations and coordination efforts. Especially when teams are under pressure, fluid team members may struggle to critically evaluate and communicate their concerns with other team members. When fluid team members cannot oppose certain processes during the development of the team’s collective understanding, critical information could be missed, leading to failure and setting the team and organization back (De Dreu, 2002). Therefore, to build resilience toward more critical evaluations when fluid team members come and go from the team, the teamwork processes present at that point in the project must be supplemented by the teamwork and cognitive processes that were developed earlier when the limitations of the idea were not as heavily considered in the vision.

Considering the nature of innovative problems and the required knowledge integration, evaluation will expand the team’s efforts to new areas of expertise. Teams that are resilient to the stressors of knowledge integration and pressure better minimize the disruptions toward team processes prior to the onset of the challenge (Alliger et al., 2015). Minimizing disruptions in innovation teams requires effective planning and reflection for potential challenges and unexpected challenges that arise and disrupt teamwork processes more (Gucciardi et al., 2018). When disruptions are minimized, the team can focus on resolving the novel problems they have been tasked with that require extensive knowledge integration and conceptual combination. Moreover, when the team is resilient to acute stressors or chronic challenges, team members can zero in on the labors of integrating knowledge, leading to more innovative solutions. Importantly, the team must anticipate these secondary teamwork challenges while planning and use the information gathered to prepare for unexpected events so more fruitful efforts can be undertaken that focus on integrating unique expertise.

Although reflection of the expertise and knowledge can improve future creative ideas, it does not suggest the team’s interpersonal processes will remain viable for that knowledge to be used effectively. Fisher (2014) elaborated on the planning and reflection to suggest teams should engage in both taskwork and teamwork planning. Importantly, team reflexivity will facilitate the sensemaking processes of previous knowledge that can allude to the knowledge that is necessary to find a creative solution. However, reflection of teamwork processes may not inform on how future teamwork will lead to more innovation when we consider the creative processes the team will engage may be contradictory to previous efforts. The sensemaking processes a team engages in to understand how the teamwork strategies influenced the team’s effectiveness will need to be more focused on developing their shared mental model. In other words, by focusing on teamwork during reflection, the diverse innovation team can understand the connection between teamwork and knowledge integration and how those combinations led to more innovation. When challenges arise that team members have encountered, the team already has an understanding of how to overcome those challenges, and more effort can be put toward the novel challenges the team is facing in this new stage of the innovative idea.

When unexpected challenges arise, team resources (e.g., social, cognitive, tangible resources) become depleted and teams can either develop or dissipate under the pressure of delivering a usable product. Teams that successfully overcome such struggles develop a stronger mental model because innovative problems requires the team to adapt to the ill-defined task and environment, or the innovative idea will fail. For innovation teams to be successful team members need to accumulate resources with each adverse event when the trajectory of cognitive and social resources of the team naturally declines throughout the creative process. Innovation teams cannot just be resilient to these challenges; they must overcome each challenge to build a stronger collective understanding and shared mental model that expands the team’s knowledge structure to include a solution for a novel problem. For teams to overcome such challenges and gain cognitive and social resources they must endure and assess the challenge quickly, accurately, and with an honest understanding of the feasibility of the idea. If the idea cannot be implemented, innovation teams need to explore refining their ideas with the current expertise or if fluid membership is necessary. This will help them to anticipate and plan for a change in team composition and subsequent social structure that is necessary for effective knowledge integration.

In the context of fluid innovation teams, reflection and planning will likely be followed by team membership change. Teams engaging in reflection, with the previous fluid team member or not, will need to make sense of previous team decisions or previous teamwork behavior. To structure the novel and complex knowledge appropriately, innovation teams can engage in debriefing to effectively sensemake before the new fluid team member joins the team. This ensures the new team member is joining a team that is aware of previous task and teamwork failures and has made changes to be more effective. Debriefing is the process in which team members discuss and interpret recent events to engage with tasks more efficiently (Allen et al., 2018; Scott et al., 2022). Debriefing is particularly useful when teams need to monitor and respond quickly to ill-defined environments where error and failure are costly (Weick et al., 2005). Through debriefing, the team can manage the changing environmental, situational, and solution-based information that affects the standards to be innovative in that domain. Through each iteration of a creative failure or success, the team can debrief and continuously update its collective understanding. When team members remain collectively updated on current structural knowledge or environmental changes they can generate more novel and useful knowledge and assess how teamwork is contributing to the success of the team.

Debriefing is imperative for effective learning if team members feel psychologically safe to engage in dissenting and nonconforming discussions (Scott et al., 2015). During debriefing sessions, the team can discuss the efficiency and productivity of regular operations but also discuss near misses and critical incidents that occurred during the shortened period of time. In discussing the specific team successes and failures throughout the innovative process, learning can compound, and the integrated knowledge becomes the new knowledge base once the team has made sense of what occurred in that moment of the idea. In other words, the shared mental model can be refined through reflexive learning for it also to be reflected in the team TMS; meaning that knowledge learned during reflection, but more explicitly during debriefing, becomes intertwined into the team’s larger knowledge structure. As the team continues through creative failures and challenges, they can add to this knowledge structure and expand access to information that could be combined with a current but incomplete idea.

Learning behavior (e.g., voicing concerns, making suggestions, and providing feedback) that extends the knowledge structure is important for debriefing but also the ongoing maintenance of an environment that is psychologically safe (Allen et al., 2018). To ensure the team’s TMS is developed alongside their shared mental model, or vice versa, reflection on the teamwork processes can help to identify where knowledge integration was interrupted that may have failed. Debriefing will facilitate more discussion around teamwork failures and account for them immediately following the event. Similarly, team members who debrief together will have worked together for an extended period where discussions around teamwork can be used to adapt future teamwork processes. These debriefing sessions will facilitate trust and a psychologically safe environment for the fluid team members when they meet a group open to novel and unique perspectives and ideas. Therefore, reflection of past collective experiences can strengthen the team’s shared mental model because new knowledge is gained from addressing previous problems and applied to future teamwork processes when the team is met with a new member.

The team’s shared mental model will improve with each adverse event when teams understand the nature of team interactions that led to a particular outcome (Mathieu et al., 2000). Accumulating shared experience and learning from team interactions can foster a “road map” between team members that provides a consensus about task work (e.g., goals, available resources, roles) but also teamwork (e.g., interpersonal interactions, communication). When teams develop a consensus about the teamwork processes necessary for task execution, they likely share a compatible, but nuanced, interpretation of changes that occurred and led to effective innovation (Cannon-Bowers et al., 1993; Mathieu et al., 2000). The similarities in how team members effectively perform tasks together will contribute to new strategies for teamwork to emerge as their shared mental models become more complex and robust to adverse events.

### Resilience and knowledge integration

When there is mutual trust between fluid and stable members, there will be more development of both collective knowledge and interpersonal relationships. Creative ideas, especially those that require multidisciplinary collaboration and systems of teams, are prone to profound rates of failure (Moenkemeyer et al., 2012). In the early stages, when the team is generating ideas, a large majority of ideas will be discarded with little consequence to the team’s resources. When teams engage in collective idea generation, they interact and consider other team members’ ideas with less critical evaluation, which helps to build mutual trust early (Linsey et al., 2011). During idea generation, the team has likely built a shared vision of the idea but has not fully considered the implementability of the ideas that were generated and combined (Strange and Mumford, 2005; Zasa et al., 2022). Therefore, the likelihood of conflict when ideas are being considered between team members declines, and mutual trust increases because team members are comfortable sharing novel ideas with the team (Farh et al., 2010; Rauniar et al., 2019). Mutual trust increases because the perception of vulnerability between team members increases when members feel their ideas are being considered as part of the team’s collective understanding or combined with other team members’ expertise (Edmondson, 2003; Gu et al., 2018). Even if the idea is ultimately discarded, team members will have developed a sense of trust between them because their interaction was shared.

The mutual trust developed when generating ideas will help teams build resilience to conflict and greater rigor when considering the limitations of implementing an idea (Farh et al., 2010; Rouse, 2020). Once the team has shifted efforts to evaluating an idea, the team can continue to build on this resilience when tangible limitations exist regarding the creative idea and interpersonal relationships between fluid and stable members. Throughout the creative process, but especially towards the project’s completion, the fluid members will need to integrate into an already dynamic and complex collective understanding provided by previous members. Even though stable members help sustain a shared vision, as the project progresses, the team’s collective understanding becomes less adaptable (Pearce and Ensley, 2004). During critical evaluation immediately prior to implementation, when a large number of resources are being considered for implementing an idea, the fluid team members’ contributions may be critical. Fluid team members’ expertise is critical for idea evaluation because their unique but expert perspective could prevent innovative failure or provide insight into the efficiency and effectiveness of implementation that could be more known to stable members.

With the assumption that failure is inevitable for innovation teams (D’Este et al., 2016), the team will experience many successful and unsuccessful ideas, both of which are an avenue to integrate knowledge and learn about the technical processes that caused the idea to fail (or succeed; Cannon and Edmondson, 2005; Oeij et al., 2018). A creative failure, in this instance, represents a failed idea, not necessarily a catastrophic failure that severely sets back the team. Reflecting and evaluating past creative failures in particular helps to diagnose the teamwork processes that contributed to the failure. Considering the immense pressure to deliver at this stage of the idea, interpersonal relationships will begin to develop and will coevolve with knowledge integration to form a stronger shared mental model.

The teamwork processes that are developed with each idea, unlike the knowledge and expertise, will impact the interpersonal relationships of team members once previous fluid members have left the team. As teams, and more specifically stable team members, experience collective failure throughout the project they can refine teamwork processes with new fluid team members that were unsuccessful previously. With each creative failure or change in fluid membership, the team can bounce back using their effective social processes and unite around the shared vision that will build resilience to unexpected problems that arise throughout the project. Therefore, knowledge integration relates to resilience when accompanied by developed and strengthened interpersonal relationships between stable and fluid team members.

*Proposition 2: Teams will become more resilient to adverse novel events when stable team members have a greater capacity to develop interpersonal relationships with fluid team members.*

### Planning

It is not enough for innovation teams to integrate knowledge that is only novel; the team must also discern how to successfully implement the idea to be considered innovative. In organizations, the creative processes (i.e., generating ideas, evaluation, implementation) occur simultaneously where solutions to major limitations have yet to be identified. As a consequence, the rhythm of teams working through innovative problems would suggest teams generate and evaluate ideas at different points in the project, potentially losing an opportunity to integrate critical knowledge that would lead to innovation. For teams to access previous ideas, their collective understanding must be structured appropriately so combinations can be reconsidered and incorporated into team members collective understanding easily if they potentially lead to successful implementation. However, considering the breadth of novel and unique expertise that both fluid and stable members contribute to the collective understanding, over a period of time it may be difficult to refer back to past ideas and evaluations that should be integrated together.

The purpose of TMSs in innovation teams is to organize the abundant and novel combinations of knowledge that are being integrated into teams collective understanding. The more knowledge that is considered for a solution, the more difficult it becomes to determine which combinations would be most effective. So, having a structured way of accessing critical novel knowledge from different points in the team’s collective understanding dictates the effectiveness of the TMS. To have access to this knowledge, teams must have an understanding of past team decision-making and evaluations to apply ideas from different time points in the creative process. Fluid members are constrained by the amount of time they spend with the team and how much they can contribute to the team’s collective understanding. Therefore, the leader or team members committed to the vision must combine past collective experiences and ideas with the ideas brought forth by fluid team members with less experience and commitment to the vision. Therefore, the stable team members provide the knowledge base for the TMS to take on the novel contributions of knowledge presented by fluid team members. While stable team members provide the base for new knowledge to be integrated into the TMS, stable members can also guide fluid team members by illustrating the shared representation that has already been determined during previous iterations of the idea.

When stable team members engage in more planning and reflection on previous decision-making, the information integrated into the team’s knowledge structure expands gradually over time and does not become overloaded with new information. Planning and reflection are also considered transition processes (Driskell et al., 2018), therefore, information being processed during that period will not be disrupted by unexpected challenges or problems with an idea the team is pursuing. Stable team members can contribute more to planning and reflection because they likely have firsthand experience with past ideas that could be considered as part of the team’s new collective understanding. Stable team members can specifically identify the causal operatives that caused the idea to fail and then transfer that knowledge to fluid members so team members can integrate their expertise more effectively in the shorter time period they are a part of the team.

However, another challenge is that previously integrated expertise, that are now a shared representation among team members may be difficult to integrate with fluid team members ideas. In other words, the composition of previous novel ideas has changed under the collective shared representation and also from the new knowledge that has been integrated up until a new idea was presented. It is important to understand the novel knowledge that is integrated into the collective understanding diminishes once the fluid team member leaves and there is no longer a consistent flow of new perspectives. Over time, the team’s collective understanding becomes more disparate from the solution that was initially integrated into the team’s collective understanding earlier in the project. Similarly, the composition change of the team has potentially been through several cycles of fluid members shifting their shared mental model and decreasing the likelihood it can be easily integrated again without the necessary expertise or fluid members.

This suggests teams need to develop a system where ideas and evaluations are organized and identifiable to future team members. In developing an organized knowledge structure that can accommodate the unique knowledge being added to it by expert team members, the team can reflect back on previous ideas that were potentially useful but were initially discarded. With respect to the long-term outcomes of the team, when unexpected problems arise, the team can mitigate the disruption through reflection of past ideas and planning to emerge with a greater understanding of the problem (Gucciardi et al., 2018) and a base to generate and integrate new knowledge.

A stronger TMS helps the team plan their work more efficiently and therefore, helps their team to resolve problems more quickly and easily (Liang et al., 1995; Moreland, 1999; Zhang et al., 2007). Similarly, when teams engage in planning it can guide team members toward goal-directed behavior (Frese and Zapf, 1996; Raabe et al., 2007; see also, Villado and Arthur, 2013; Fisher, 2014). As teams pursue and engage with other creative solutions, the teams’ goals may shift; if this decision was made strategically by the team or leadership it would ultimately lead to a creative solution. However, if teams aimlessly generate ideas and pursue them without consideration of the underlying purpose of the project, team members will not be able to build efforts that, in combination, are innovative. While planning and considering the long-term goals of the project explicitly, teams can predict the tasks that will be necessary to achieve the desired outcome. Prior to implementing the idea, the team can identify these tasks and subsequent behaviors that will lead them to the next phase of the project. At this point in planning, the team can determine if the idea has underlying constraints that were not identified in earlier evaluations from the different perspectives of team members without sacrificing resources.

They can also determine if fluid membership is necessary for the next phase of the project, leading to easier cognitive integration when the team member joins the team. Proper planning relates to effective cognitive integration, following social integration, because certain characteristics of the ill-defined idea may be identified that connect to a specific combination of expertise necessary for innovation. Identifying the combinations of tasks and knowledge that will likely result in successful implementation will help to determine if implementation is possible or if fluid membership is necessary. When there is little consequence for new combinations of knowledge, ideas can be further defined and can be evaluated more deeply, improving the team’s collective understanding of the different combinations of knowledge even if the idea is not implementable.

### Team reflexivity

Reflection is a critical counterpart to planning and can be looked at as a learning opportunity to adapt cognitive and teamwork processes (Ellis and Davidi, 2005; Crowe et al., 2017). When team members reflect on past behavior or collective experiences, they can link them to particular performance outcomes. Whether the idea was a creative success or failure, team members will indicate if and how the teamwork behaviors or combinations of knowledge intended to be innovative contributed to its successful implementation. Innovation teams can also attribute success or failure events with a causal explanation of the processes associated with the outcome. This way, teams can reflect on how the orientation of team members’ knowledge and how they engage with team members is contributing to or hindering innovation. They can then use this information to reflect on the cognitive and teamwork processes that contributed to the success or failure of that idea, and adapt these processes to be more suited for creativity and innovation for the next phase of the project.

Teams develop a more robust knowledge structure when they reflect on the cognitive processes attributed to their success or failure. When teams link specific knowledge to potential outcomes, their knowledge structure develops more systematically, and if team members do so collectively it can lead to greater levels of knowledge integration. As a result, the chance of optimizing and spending valuable resources on a system or process that need not be part of the overall creative idea is mitigated because more perspectives and ideas are reflected throughout the project.

Reflection makes it easier for teams to identify past teamwork experiences that have been integrated into the team’s shared mental model as the cognitive processes have evolved. While reflecting on cognitive processes can help to structure knowledge more effectively, reflecting on the teamwork processes can be more impactful for innovative outcomes (Thayer et al., 2018). Teams who reflect on past experiences together can address the teamwork outcomes using their diverse perspectives to consider alternative explanations for why a particular outcome occurred. These explanations can spark alternative action because new ideas and observations have emerged that highlight aspects of implementation that will lead to the creative idea being unsuccessful. Importantly, considering the nature of the innovative task and the environment, alternative action plans will be necessary to integrate knowledge at a higher level. Specifically, alternative explanations or action plans may contribute to higher-order knowledge integration that can overcome the limitations of the environment or situation. Creating highly integrated concepts and novel solutions to then engaging in deep reflection and evaluation influences the creative performance of the team at each stage. This would suggest it is not enough to integrate the ideas of different team members, but actively pursue novel information that is derived from these combinations is how teams turn creative ideas into innovation.

Reflection is important at the beginning stages of the project because team members are generating a plethora of ideas, that will likely be discarded but could lead to an action plan or alternative action that is more efficient. These ideas can be referred back to at a later stage of the project where team members can derive a novel solution from a previous idea instead of exhausting resources to generate new ideas. While teams are generating ideas, team members are more likely to pursue an alternative plan than when they are evaluating the idea (Alexander and Van Knippenberg, 2014). Therefore, if the team can reflect and identify important causal factors with the ideas generated, they can consider a wider range of potential knowledge combinations that may be successful. When teams consider a wider range of perspectives earlier in the project, they can use them to pursue novel information when the team is collectively and critically evaluating the idea. This is possible because the team’s collective understanding while generating ideas, rather than evaluating them, is more malleable and open to alternative pathways to innovation.

As the team reflects on past collective experiences, they can connect and integrate them to future implementation planning. Teams who reflect and identify causal relationships that either resulted in failure or success can connect these causes to planned collective tasks, preventing a similar outcome from occurring again or improving the current process. This notion is especially important when teams are being challenged to comprehensively assimilate information that is likely conflicting but necessary to generate novel solutions and implement them effectively. For this reason, planning and subsequent reflection, or vice versa, can act as a guide for teams to consider a more efficient path to successful integration and assimilation of knowledge. The idea that more immersive planning and reflection can also be applied to teamwork processes has been shown to be more important than the planning of cognitive tasks to develop social processes and interpersonal relationships that emerge with cognitive integration (Thayer et al., 2018). Effective planning relates to team reflexivity through the coevolution of cognitive and social processes, suggesting both are necessary for effective team innovation.

*Proposition 3: Planning will increase the degree of team reflexivity, allowing fluid and stable team members to connect diverse expertise to certain innovative outcomes.*

### Resilience and team reflexivity

A team that engages in reflexive behavior will have structured and organized access to previous knowledge and current ideas that are being integrated into the team’s collective understanding. This effect can be strengthened if the team can engage in effective planning prior to each phase of the project followed by team reflection. The cycle of planning and reflection, likely accompanied by task and membership change, can help stable members anticipate obstacles of fluid membership, the necessity of expert knowledge, unexpected problems that could arise, and task change while also mitigating the disruptions of the teamwork processes that affect the team delivering a usable product. When teams separate and then reconnect specific processes with team outcomes, team members can begin to differentiate the processes that do and do not contribute to innovation. When the processes and outcomes are connected, stable members and the leader can adapt and sustain the team’s shared mental model while experiencing task and membership changes. Cognitive resilience for team members to integrate knowledge also improves because information and expertise have been organized systematically and with consideration that the idea may be useful at a later point in the project. As a result, the connected knowledge and outcomes are structured prior to implementation and are identifiable when the team comes across a similar problem.

The more interdependent the task between team members stimulates the development of the TMS (Wegner et al., 1991; Moreland, 1999; Hollingshead, 2001; Brandon and Hollingshead, 2004). Innovative endeavors are challenging and complex, which works with the TMS because interdependence is necessary, and more knowledge is distributed across the team (Zhang et al., 2007). When knowledge expands to other team members, more unique knowledge combinations arise, but team members also become aware of others’ skills and coordinate their expertise to complete the task. The innovation teams’ TMS, which helps access and coordinate knowledge, will improve with planning and reflection (Peltokorpi, 2008). Planning and reflection further break down each step of generating, evaluating, and implementing the idea with careful consideration of the knowledge and skills that each member possesses. For systems of teams who are working on many different aspects of the project simultaneously, understanding the integration that is necessary for generally separate ideas can also be improved through planning and reflection. Therefore, the stable members can build their TMS with each cycle of team membership, which will lengthen the amount of time the team considers ideas that could potentially be integrated. This may improve knowledge integration even further because fluid team members who were not present during that phase of the project, even if several iterations from the current collective understanding, have access to this information that would otherwise not have been effectively considered using their expertise.

This will also improve team planning and reflexivity toward team goals, teamwork strategies, and processes that are effective and should be reconsidered for the next phase of the project. Teamwork processes, in particular, that are planned throughout the life cycle of the team will elicit a stronger mental model as the team is carrying out the creative idea. As the team progresses, teamwork skills can help to withstand the adverse effects of fluid membership and task change because stable team members have experience with a wider range of team members. With this, they become accustomed to the difficulty and complexity of integrating knowledge with unfamiliar team members and in an ill-defined and unforgiving environment. When the team can overcome fluidity along with the teamwork necessary to integrate knowledge, they can generate novel solutions that exceed the threshold for innovation. This would suggest that team reflexivity and planning relate to resilience based on the ability of the team to connect team-level action to specific team outcomes.

*Proposition 4: Innovation teams will become more resilient to fluid membership and complex, innovative problems when teams effectively plan and reflect through each phase of the project.*

### Resilience and innovation

Failure, followed by extensive evaluation, can lead to more reflection on why the idea was not successful. Effective planning and reflection are rooted in the ability of the team to integrate knowledge and generate novel solutions that require implementation. Teams are motivated to see their creative ideas come to fruition because of their shared vision and commitment to achieving innovative success. Therefore, team members are willing to engage in difficult and complex knowledge integration and complex problem solving that requires a team to effectively disentangle contradictory information - often involving and only possible with extensive planning and reflection.

Resilience is developed over time but, more importantly, is improved when the team experiences several creative failures, near misses, and critical incidents while the team is developing the idea. To build resilience towards critical incidents during implementation, the team must experience creative failure together and reflect on the knowledge integration processes that resulted in the innovative failure or success. When the team becomes more resilient to unexpected problems, the team can restructure and coordinate expertise toward a solution while collectively planning. Coordination is a critical factor for team resilience when complicated problems require a specific combination of expertise to be resolved (Gomes et al., 2014). This effect is stronger for fluid teams because the amount of diverse expertise that is available expands, increasing the likelihood of innovation. This also references the necessity of planning and reflection in fluid teams, considering the depth and complexity of expert knowledge that is streamed into the team’s collective understanding along with the novel knowledge that is derived from integrating this expert knowledge. For fluid teams, there is an emphasis on coordination because the requirements for implementing the idea far exceed the resources and effort necessary to generate and integrate knowledge. In other words, fluid teams need to coordinate their actions because of the precariousness of the knowledge and expertise that are integrated when the team is finding an innovative solution. Therefore, for the team to achieve innovation, they need to implement the idea without error at each phase of development, requiring expertise from many resources. Successful innovation becomes more likely as the team progresses in the project because they can coordinate expert knowledge and integrate it since a larger portion of the project is complete but also because the interpersonal relationships have developed.

If the innovation team’s coordination is affected by fluid membership or unexpected issues later in the team’s development, they are more resilient because some structure has already been facilitated in the planning process and by the status of the project. When teams plan, there is a greater opportunity to develop a new course of action and adapt to unanticipated or anticipated problems. Both planning and reflection contribute to the team’s collective understanding more broadly while also contributing to the success of the idea.

*Proposition 5: Team resilience will increase the efficiency of innovation teams’ knowledge integration and team reflexivity leading to the discovery of innovative solutions.*

### Fluid membership and team resilience

The degree of fluid membership will depend on the nature of the problem and should be considered as part of the implementation. When innovation teams are stable, communication can become lax and lead to errors (Bushe and Chu, 2011). In high-reliability organizations where the consequences of error are severe, team members must be vigilant toward miscommunication to prevent error. With new members, stable team members need to communicate with a higher degree of precision to ensure they are operating safely and avoiding erroneous mistakes because they are unfamiliar with the domain. As the team becomes more familiar and less fluid, their communication lines weaken, leading to more error-prone communication and less effective knowledge sharing (Fodor and Flestea, 2016). When teams do not effectively communicate, the amount of unique knowledge shared no longer passes through different expert team members. Therefore, team members will be unable to coordinate the activities necessary to implement the novel solution, whether generating and integrating ideas or evaluating these combinations for their usefulness.

On the other hand, teams with a high level of fluidity will have difficulty adjusting to new members who are cycling in and out of the team. The influx of new members, especially in smaller innovation teams, will severely disrupt the social processes required for knowledge integration (Huckman and Staats, 2011; Fodor and Flestea, 2016). In particular, stable members will not have the capacity to interact and develop relationships with fluid team members when the number of fluid members increases or if the length of time they are on the team is reduced. When fluid and stable members do not effectively socially integrate, the likelihood of conflict increases because they are less familiar with that team members’ teamwork and cognitive processes. Since conflict weakens the degree of psychological safety in innovation teams, it may adversely affect the degree to which team members feel comfortable sharing information and ideas and engaging in intensive knowledge integration.

Similarly, this may negatively impact knowledge integration because the flow of new knowledge exceeds what the team can integrate into their collective understanding. Therefore, the team becomes overloaded with information, and it is difficult to determine which ideas should be integrated or have the highest chance of being innovative. As a consequence, more knowledge that could be highly impactful will not be considered as part of the team’s collective understanding. To mitigate the loss of knowledge with each fluid membership cycle, innovation teams can develop processes where the appropriate amount of knowledge can be used to stimulate knowledge integration but not disrupt teamwork processes. Taken together, fluid membership relates to innovation such that too much or little fluidity results in disruptions to the social processes that facilitate knowledge integration.

*Proposition 6: The relationship between fluid membership change and team resilience will be curvilinear, where teams with a low and high rate of membership change will be the least resilient.*

## Future directions

Our framework takes into consideration how the team’s knowledge structure develops to make knowledge more accessible and to enhance knowledge integration between team members. The relationships between mutual trust and knowledge integration and plan formulation and team reflexivity on team innovation have already been established in the literature. However, we have little empirical knowledge of the interaction between knowledge integration and team reflexivity and its effects on team resilience and innovation. In testing these relationships empirically under the context of fluid membership, the theoretical and empirical literature would benefit from measuring these variables across time.

Researchers will find value in measuring these relationships across time to assess the compounding effects of knowledge integration and team reflexivity on resilience. Understanding how knowledge integration develops over time will help determine if knowledge integration is impacted by the new knowledge offered by fluid team members. Knowledge integration, along with team reflexivity, will help to explain how teams can effectively integrate knowledge with each point of introspection and if it helps to structure and organize knowledge to a greater degree. The propositions (e.g., Proposition 5) can be further confirmed by addressing team reflexivity and knowledge integration in fluid teams by illustrating when new knowledge is added and eventually integrated into the team’s knowledge structure systematically; it will result in higher levels of resilience, leading to more novel solutions as the team moves through the innovation process.

## Conclusion

The recent desire to achieve more groundbreaking innovative goals has created a need to understand how innovation teams engage in teamwork to produce innovative knowledge. The nature of innovative work is inherently apt to failure and requires a symbiotic understanding of specialized expertise that overcomes the complexity of the task and teamwork processes that contribute to knowledge integration. It is necessary to understand the processes innovation teams engage in to understand how novel knowledge is developed and built upon where a functional and usable product is successfully implemented. Similarly, it is important to consider the dueling processes innovation teams must engage in to successfully conceptualize the problem and environment, generate novel solutions, and implement the idea successfully. To address the nature of team innovation and the challenges teams are required to overcome to be successful, we developed a framework that considers resilience as a mechanism to build the cognitive and teamwork processes necessary for the innovative idea to reach a successful conclusion.

We propose the conduit for innovation teams to build resilience is through a combination of effective knowledge integration and team reflexivity. Considering the duality of these constructs suggests teams need to generate an extensive compilation of ideas whose connections are not obvious but imperative for generating new knowledge. Working with these generated ideas, teams need to identify an effective strategy for implementation and follow through for the idea to be useful. We argue that effective planning and reflection can help the team to identify the characteristics of the idea that caused it to fail and prevent a similar situation from happening later in the project. We further the argument for organizing the team’s knowledge structure through planning and reflection by considering it a system where teams can derive cognitive concepts and refine teamwork processes that will lead to innovation over time. Most importantly, the ability of the team to build integrated knowledge and refine teamwork processes to be more efficient will lead to innovation, even if the team was affected by an adverse event in ascertaining the innovative solution.

We also considered an inherent need for innovation teams to remain fluid, especially during times when crucial decisions are being made that will impact the effectiveness of later efforts. Fluid membership is considered a balancing act where teams constantly encounter the adverse effects of unfamiliar team members but engage with them to integrate useful knowledge. Ultimately, fluid membership is a catalyst for innovation and is considered a valuable resource that innovation teams harness when the current collective knowledge cannot overcome innovative challenges. Fluid membership, while advancing knowledge within the team, can also help to develop the teamwork processes that are needed to overcome crucial challenges of the project. We conclude that fluidity in the context of innovation teams can challenge team members to embrace counterintuitive perspectives of expert team members and find unique ways to incorporate their knowledge into the creative idea. However, this is contingent on developing stronger interpersonal connections in innovation teams so these perspectives can be integrated to the level necessary for innovation.

## Data availability statement

The original contributions presented in the study are included in the article/supplementary material, further inquiries can be directed to the corresponding authors.

## Author contributions

RL: Writing – original draft. ES: Writing – review & editing.
